# Relationship of serum vitamin D levels with coronary thrombus grade, TIMI flow, and myocardial blush grade in patients with acute ST-segment elevation myocardial infarction

**DOI:** 10.1186/s43044-020-00118-5

**Published:** 2020-11-23

**Authors:** Abdallah Ahmed Abdallah, Mohamed Ahmed Abd Elrhman, Ahmd Elshazly, Islam Bastawy

**Affiliations:** Department of Cardiology, Ain Shams University, Cairo, Egypt

**Keywords:** Interventional cardiology, No-reflow, Vitamin D, Acute myocardial infarction

## Abstract

**Background:**

Vitamin D deficiency is a prevalent condition that is found in about 30–50% of the general population, and it is increasing as a new risk factor for coronary artery disease. Our study aimed to evaluate the relationship of serum vitamin D levels with coronary thrombus burden, Thrombolysis In Myocardial Infarction flow grade, and myocardial blush grade in patients managed by primary percutaneous coronary intervention for their first acute ST-segment elevation myocardial infarction.

**Results:**

Eighty patients were included in the study with their first acute ST-segment elevation myocardial infarction and were managed by primary percutaneous coronary intervention. According to the serum concentrations of vitamin D, the study population was divided into 2 groups: group A with abnormal vitamin D levels less than 30 ng/ml (50 patients) and group B with normal vitamin D levels equal to or more than 30 ng/ml (30 patients). Angiographic data was recorded before and after coronary intervention. On comparing thrombus grade and initial and post-procedural Thrombolysis In Myocardial Infarction flow between both groups of patients, there was no significant difference (*p* = 0.327, *p* = 0.692, *p* = 0.397). However, myocardial blush grade was better in patients with normal vitamin D levels (*p* = 0.029) without a significant correlation between vitamin D concentration values and myocardial blush grade (*r* = 0.164, *p* = 0.146).

**Conclusions:**

Patients with first acute ST-segment elevation myocardial infarction and normal vitamin D levels undergoing primary percutaneous coronary intervention had better myocardial blush grade and more successful microvascular reperfusion in comparison with patients with abnormal vitamin D levels. There was no significant difference between the normal and abnormal vitamin D groups regarding the coronary thrombus grade and Thrombolysis In Myocardial Infarction flow.

## Background

Despite the medical and technological improvements in the re-vascularization procedures in coronary artery diseases (CAD), ST-segment elevation myocardial infarction (STEMI) remains a significant health concern. Primary percutaneous coronary intervention (PCI) is the favorable treatment option for restoring perfusion to the affected area of the myocardium as soon as possible. In STEMI, the incidence of no-reflow has been reported to be ranging between 11 and 41%, depending on several clinical and angiographic factors in addition to the adopted definition of no-reflow [1]. The pathogenesis of no-reflow is known to be multi-factorial, and its possible mechanisms include injury related to ischemia, reperfusion, endothelial dysfunction, microvascular spasm, and distal thromboembolism [2].

Vitamin D deficiency causes endothelial dysfunction through its direct or indirect effect through the upregulation of the renin-angiotensin system or via induction of smooth muscle proliferation and a pro-inflammatory state [3]. The prevalence of vitamin D deficiency was estimated to be approximately 30–50% of the general population [4], and several studies have shown an association between vitamin D deficiency and cardiovascular diseases (CVD) including hypertension (HTN), acute myocardial infarction (AMI), heart failure, CAD, metabolic syndrome, and diabetes mellitus (DM) [5]. This study aimed to evaluate serum vitamin D level’s relationship with coronary thrombus grade, Thrombolysis In Myocardial Infarction (TIMI) flow, and myocardial blush grade (MBG) in patients with first acute STEMI undergoing primary PCI.

## Methods

This was a prospective cohort study that included 80 patients with their first acute STEMI who presented within 12 h after the onset of chest pain and referred for primary PCI in the period between December 2017 and November 2018.

Patients were excluded from the study if they had a previous history of ischemic heart disease (prior AMI, PCI, or coronary artery bypass graft), active infection, or malignancy. Patients on previous anti-thrombotic or anticoagulant medications and patients who received thrombolytic therapy before coronary angiography were also excluded from the study.

We applied the European Society of Cardiology guidelines for STEMI management in diagnosis [6]. A detailed history was taken, and a full clinical examination was done searching for CAD risk factors including age, sex, positive family history of premature CAD, body mass index, smoking, dyslipidemia, HTN, and DM.

Patients with DM were identified on admission as HbA1c ≥ 6.5% (48 mmol/mol) or fasting plasma glucose ≥ 7.0 mmol/l (126 mg/dl) or 2-h postprandial glucose ≥ 11.1 mmol/l (≥ 200 mg/dl) or those using either oral hypoglycemic agents or insulin treatment [7]. Dyslipidemia was defined as total cholesterol of at least 200 mg/dl or using anti-hyperlipidemic therapy on admission [8]. HTN was defined as office systolic blood pressure values ≥ 140 mmHg and/or diastolic pressure values ≥ 90 mmHg or using antihypertensive therapy on admission [9].

All patients received aspirin 300 mg and clopidogrel 600 mg (available P2Y12 inhibitor at our institute) before their transfer to the catheterization laboratory. Emergency coronary angiography was performed using the percutaneous femoral or radial approach. Heparin (100 U/kg) was administered in all patients during the primary PCI procedure [10].

The crossing of the occluded culprit artery was done using various guide wires, and pre-dilatation was performed via balloon angioplasty if necessary. Routine stenting using a drug-eluting stent was attempted directly or following balloon angioplasty. The usage of thrombus aspiration catheter and administration of tirofiban infusion were chosen according to the decision of the interventional cardiologist [11]. Thrombus grading was done using the TIMI thrombus scale grade [12].

The initial TIMI flow was assessed at the beginning of the procedure before wire crossing and the final TIMI flow immediately after the primary PCI using the TIMI flow grade classification [13]. The MBG was assessed visually on the angiogram and interpreted immediately after the primary coronary angioplasty procedure by the performing cardiologist: grade 0, no myocardial blush; grade 1, minimal myocardial blush; grade 2, moderate myocardial blush; and grade 3, normal myocardial blush [14].

Ejection fraction (EF) was measured within the first 48 h after primary PCI by the modified Simpson’s method using a (Vivid S5, General Electric Healthcare) echocardiography device.

We obtained peripheral venous samples on admission from all patients before the primary PCI then they were centrifuged early after collection, and extracted serum was stored at − 20 °C. We measured 25-hydroxy vitamin D concentrations (vitamin D) using the enzyme-linked immunosorbent assay (Calbiotech, 25(OH) Vitamin D ELISA).

According to serum vitamin D levels, study population was divided into 2 groups: group A, which included patients with abnormal vitamin D levels less than 30 ng/ml, and group B, which included patients with normal vitamin D levels equal to or more than 30 ng/ml.

### Statistical analysis

We used version 20.0 of the Statistical Package for Social Sciences (SPSS Inc., Chicago, IL, USA) in the data analysis. Quantitative data was expressed as mean ± standard deviation. Qualitative data was expressed as frequency and percentage. Comparing two means was done using the independent-samples *t* test, the chi-square (*χ*^2^) test was used in comparing proportions between qualitative parameters, and correlation between 2 sets of variables was done using the Spearman correlation coefficient (*r*) test. The confidence interval was set to 95, and the accepted margin of error was set to 5%. So, the *p* value was considered significant as follows: probability (*p* value) < 0.05 was considered significant, and *p* value > 0.05 was considered non-significant.

## Results

Abnormal vitamin D levels (< 30 ng/ml) were found in 50 patients representing group A while normal vitamin D levels (≥ 30 ng/ml) were found in 30 patients representing group B.

### Baseline characteristics and clinical data

There was no significant difference between both groups on comparing baseline characteristics and clinical data including age, gender, CAD risk factors, pain to door (PTD) time, door to balloon (DTB) time, and Killip class. Also, there was no significant difference between both groups on comparing EF measured within the first 48 h from primary PCI. This data was summarized in Table 1.

**Table 1:** Comparing baseline characteristics and clinical data between both groups

Demographic data	Group A (vit. D < 30) (no. = 50)	Group B (vit. D > 30) (no. = 30)	***p*** value
**Age (years)**	54.82 ± 7.55	53.60 ± 7.07	0.476
**Sex**			
Female	13 (26.0%)	8 (26.7%)	0.948
Male	37 (74.0%)	22 (73.3%)	
**Smoking**	18 (36.00%)	8 (26.70%)	0.388
**HTN**	16 (32.0%)	10 (33.3%)	0.902
**DM**	14 (28.0%)	8 (26.7%)	0.897
**BMI (kg/m**^**2**^**)**	26.62 ± 2.59	26.70 ± 2.93	0.899
**Dyslipidemia**	9 (18.0%)	5 (16.7%)	0.879
**Positive family history**	8 (16.0%)	6 (20.0%)	0.649
**PTD (h)**	7.500 ± 2.39	6.67 ± 3.08	0.210
**DTB (min)**	73.00 ± 9.15	74.33 ± 11.20	0.564
**EF%**	42.26 ± 7.48	41.97 ± 7.76	0.867
**Killip class**			
I	44 (88.0%)	27 (90.0%)	0.738
II	5 (10.0%)	3 (10.0%)	
III	1 (2.0%)	0 (0.0%)	

*HTN* hypertension, *DM* diabetes mellitus, *BMI* body mass index, *PTD* pain to door, *DTB* door to balloon, *EF* ejection fraction

### Angiographic data

**Fig. 1:**
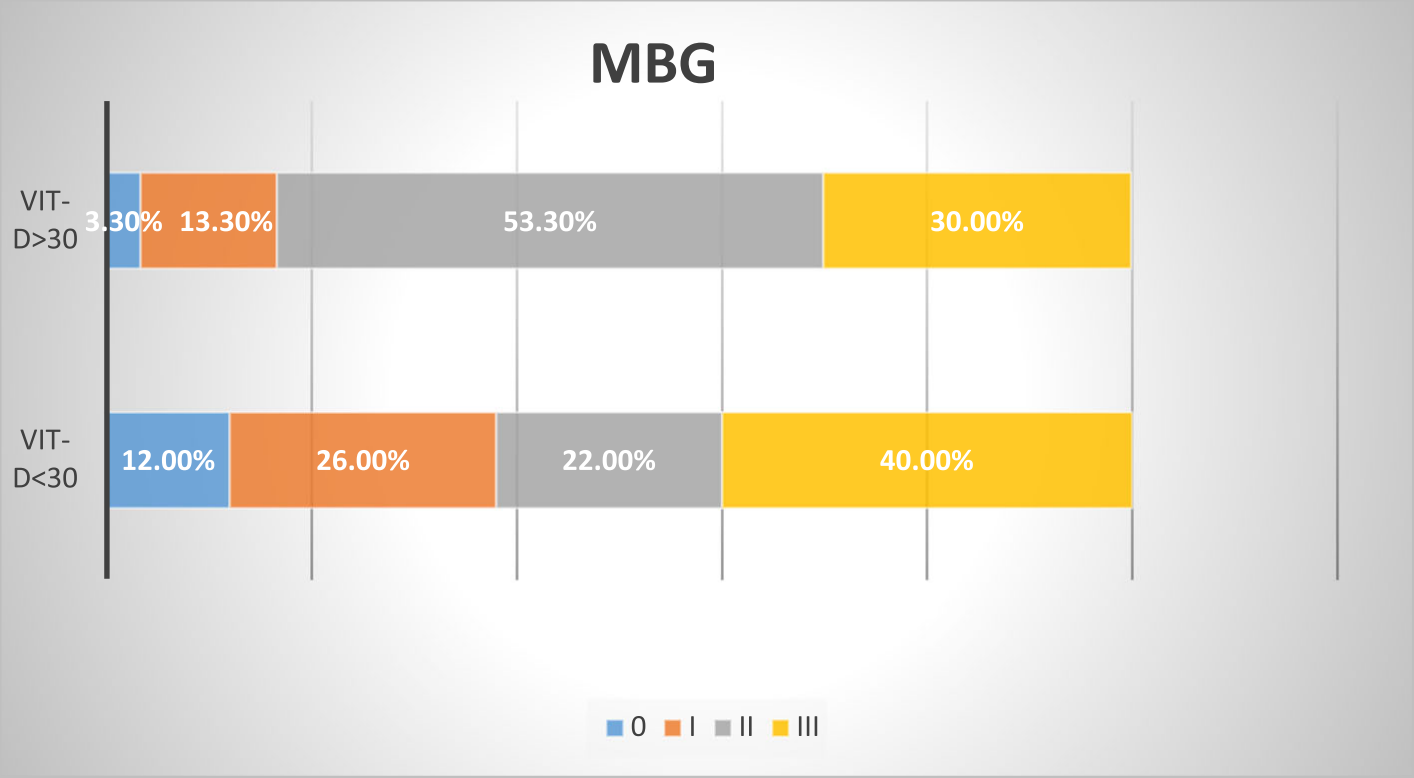
Comparing MBG between both groups

A comparison between both groups regarding the initial angiography, final angiography, and interventional data was shown in Table 2. On comparing the number of affected vessels, culprit vessel, thrombus grade, initial TIMI flow, use of thrombus aspiration, or tirofiban infusion between both groups, we did not find any significant difference. Also, there was no significant difference in comparing post-procedural TIMI flow. However, there was a significant difference between both groups on comparing post-procedural MBG (Fig. 1), which was better within group B patients with normal vitamin D levels (*p* = 0.02). Group A patients had MBG III in 20 patients (40%), MBG II in 11 patients (22%), MBG I in 13 patients (26%), and MBG 0 in 6 patients (2%) while group B patients had MBG III in 9 patients (30%), MBG II in 16 patients (53.3%), MBG I in 4 patients (26%), and MBG 0 in 1 patient (3.3%) (*p* = 0.029). This significant difference was maintained when we compared the success of myocardial reperfusion between both groups. Patients with MBG II or III represented successful myocardial reperfusion, while patients with MBG 0 or I represented unsuccessful myocardial reperfusion. Successful myocardial reperfusion was achieved in 31patients of group A (62%) versus 25 patients of group B (83.3%) (*p* = 0.047). However, on correlating serum vitamin D concentration values to MBG, it was found to be non-significant (*r* = 0.164, *p* = 0.146).

**Table 2:** Comparing the initial angiography and interventional data between both groups

Angiographic and interventional data	Group A (vit. D < 30) (no. = 50)	Group B (vit. D ≥ 30) (no. = 30)	***p*** value
**Initial TIMI flow**			
0	31 (62.0%)	15 (50.0%)	0.692
I	6 (12.0%)	4 (13.3%)	
II	7 (14.0%)	7 (23.3%)	
III	6 (12.0%)	4 (13.3%)	
**Thrombus grading**			
1	0 (0.0%)	2 (6.7%)	0.327
2	6 (12.0%)	5 (16.7%)	
3	7 (14.0%)	3 (10.0%)	
4	6 (12.0%)	5 (16.7%)	
5	31 (62.0%)	15 (50.0%)	
**Number of vessels**			
Single vessel	39 (78.0%)	24 (80.0%)	0.832
Multi-vessels	11 (22.0%)	6 (20.0%)	
**Culprit vessel**			
LAD	31 (62.0%)	18 (60.0%)	0.984
RCA	11 (22.0%)	7 (23.3%)	
LCX	8 (16.0%)	5 (16.7%)	
**Thrombus aspiration**	3 (6.0%)	0 (0.0%)	0.171
**Tirofiban infusion**	15 (30.0%)	5 (16.7%)	0.182
**Post-PCI TIMI flow**			
I	4 (8.0%)	1 (3.3%)	0.397
II	11 (22.0%)	4 (13.3%)	
III	35 (70.0%)	25 (83.3%)	
**MBG**			
0	6 (12.0%)	1 (3.3%)	0.029*
I	13 (26.0%)	4 (13.3%)	
II	11 (22.0%)	16 (53.3%)	
III	20 (40.0%)	9 (30.0%)	
**Myocardial reperfusion**			
Unsuccessful (MBG 0, I)	19 (38.0%)	5 (16.7%)	0.047*
Successful (MBG II, III)	31 (62.0%)	25 (83.3%)	

* = significant

*TIMI*Thrombolysis In Myocardial Infarction, *MBG* myocardial blush grade

## Discussion

Coronary revascularization is the main target of primary PCI for acute STEMI which is a life-threatening condition that requires fast diagnosis and proper intervention. However, a considerable number of patients still develop a no-reflow phenomenon during primary PCI having a worse outcome [15]. Several studies showed that vitamin D deficiency was prevalent in AMI patients, and they indicated worse STEMI outcomes in those patients with vitamin D deficiency [16, 17].

### Abnormal vitamin D levels and myocardial infarction

Our study showed that patients with acute STEMI had a higher prevalence of abnormal vitamin D levels in comparison with the general population as abnormal vitamin D levels (insufficient or deficient) were found in 62.5% of the study population. This observation is supported by the study of Lee and his colleagues in 2011 that showed even higher rates of abnormal vitamin D levels in AMI patients as 96% had abnormal vitamin D levels [18]. Also, Mahdavi and his colleagues in 2013 have observed vitamin D deficiency in most of the patients with acute coronary syndrome, and normal vitamin D levels were found only in 10.6% of patients [19]. Similar results were presented by Correia and his colleagues in 2013 in their study that mentioned that vitamin D levels were normal only in 10% of patients with AMI [20].

### Abnormal vitamin D levels and angiographic findings

We did not find in our study a significant difference between both groups regarding the number of affected vessels which is matching with the study conducted by Goleniewska and his colleagues in 2013 that did not find a significant difference in vitamin D levels among STEMI patients with single or multi-vessel affection [21].

It is important to mention that combining post-procedural TIMI flow and MBG is more accurate in the angiographic assessment of no-reflow as post-procedural TIMI flow III reflects successful epicardial reperfusion that is not always associated with successful micro-vascular or myocardial tissue reperfusion that is achieved by having MBG II or III [22, 23]. Despite the restoration of epicardial flow in a considerable number of patients (TIMI III flow), impaired myocardial reperfusion (MBG 0 or I) is associated with poor outcomes [24].

Our study did not find a significant difference in comparing coronary thrombus grade and initial or post-procedural TIMI flow between both groups. However, MBG was significantly higher in patients with normal vitamin D levels, but there was no significant linear correlation between vitamin D levels and MBG. Cerit and his colleagues in 2019 conducted a study that showed that the mean values of vitamin D levels were lower in patients who developed no-reflow; however, vitamin D values were not shown to be an independent predictor of no-reflow that was defined by achieving TIMI flow less than III [25]. This may point to a potential role of abnormal vitamin D levels in the reduction of successful reperfusion at the microvascular level rather than affecting epicardial coronary thrombus grade or epicardial reperfusion. What supports our hypothesis is that high residual platelet reactivity measured during primary PCI is associated with more incidence of no-reflow [26], and patients with vitamin D deficiency in the study of Verdoia and her colleagues in 2016 had higher residual platelet reactivity with adenosine diphosphate (ADP) when measured 30–90 days after coronary intervention in patients kept on dual antiplatelets raising concerns that vitamin D deficiency may impair ADP antagonists’ effectiveness and it may have a potential role in increasing risk of clopidogrel resistance [27].

On the other hand, our explanation of the absence of a significant linear correlation between vitamin D concentration values and MBG is that the clinical importance of MBG relies on being either MBG 0 or I versus MBG II or III rather than on the exact MBG. And, in our study, group A with abnormal vitamin D levels had a higher percentage of MBG III. However, on comparing the overall success of microvascular reperfusion, it was significantly higher in group B with normal vitamin D levels.

Moreover, a study conducted by Şen and his colleagues in 2017 that evaluated the association between vitamin D levels and grade of collateralization in STEMI patients found that vitamin D levels were higher among patients with well-developed collaterals that are dependent on various endogenous mediators such as vascular endothelial growth factor, nitric oxide, and other neuro-humoral markers [28].

### Abnormal vitamin D levels and left ventricular ejection fraction

Despite achieving better myocardial reperfusion in patients with normal vitamin D levels, we did not find a significant difference between both groups in EF measured within the first 48 h after primary PCI, which is consistent with the findings of a study conducted by Khalili and his colleagues in 2012 that did not find an association between LV systolic function and vitamin D levels [29]. This may be due to early assessment of LV functions after AMI within the first 48 h where myocardial stunning is found. However, later assessment of LV function may show a difference as shown in the study of Rehman and his colleagues in 2020 that showed significantly higher LV EF in STEMI patients with higher MBG when LV function was assessed 3 months after the primary PCI [30].

### Study limitations

This was a single-center study, and further studies with a larger number of patients are needed to confirm our findings and to assess minor differences seen in thrombus grading between both groups. Also, our population included only one geographical region. It is also important to mention that vitamin D status can change with various factors, such as seasonal variation, geography, latitude, and sunlight exposure.

## Conclusion

Patients with first acute STEMI and normal vitamin D levels undergoing primary PCI had better MBG and more successful microvascular reperfusion in comparison with patients with abnormal vitamin D levels. There was no significant difference between normal and abnormal vitamin D groups regarding the coronary thrombus grade and TIMI flow.

## Data Availability

The datasets used and analyzed during the current study are available from the corresponding author on reasonable request.

## Abbreviations

ADP: Adenosine diphosphate
AMI: Acute myocardial infarction
BMI: Body mass index
CAD: Coronary artery disease
CVD: Cardiovascular diseases
DM: Diabetes mellitus
DTB: Door to balloon
EF: Ejection fraction
HTN: Hypertension
LAD: Left anterior descending artery
LCX: Left circumflex artery
LV: Left ventricular
MBG: Myocardial blush grade
PCI: Percutaneous coronary intervention
PTD: Pain to door
RCA: Right coronary artery
STEMI: ST-segment elevation myocardial infarction
TIMI: Thrombolysis In Myocardial Infarction
